# Bell’s Palsy Unmasked: A Compelling Case Study of Facial Nerve Palsy During Pregnancy

**DOI:** 10.7759/cureus.51369

**Published:** 2023-12-30

**Authors:** Dharmesh J Patel, Kamlesh Chaudhari, Deepti Shrivastava, Apoorva Dave

**Affiliations:** 1 Department of Obstetrics and Gynaecology, Jawaharlal Nehru Medical College, Datta Meghe Institute of Higher Education and Research, Wardha, IND

**Keywords:** management strategies, obstetric neurology, corticosteroids, immune system changes, bell`s palsy

## Abstract

Facial paralysis occurs more frequently in pregnant individuals, affecting them two to four times more often than those who are not pregnant, making it the most frequent unilateral cranial nerve pathology in pregnancy. This case report describes a 29-year-old primigravida's presentation, examination, and treatment of left-sided (unilateral) facial nerve palsy during 32 weeks of gestation. Concerns regarding possible underlying reasons were raised when the patient suddenly developed left-side facial weakness. We examined her history, clinical assessment, and diagnosis methods, which included laboratory and neuro-imaging tests. The difficulties of managing this illness during pregnancy are explored, taking into account the well-being of the developing fetus and mother. There are several causes for facial nerve palsy during pregnancy, including idiopathic causes, vascular problems, and viral infections. Here, we emphasize the value of a multidisciplinary approach comprising obstetricians, neurologists, and other medical professionals to guarantee the best possible care. The paper also underscores the necessity for prompt diagnosis and suitable interventions to reduce problems and foster a successful outcome. This case report adds to the limited literature on facial nerve palsy in pregnancy by highlighting individualized medical care and teamwork in addressing this uncommon but serious condition.

## Introduction

Bell's palsy (BP), also known as idiopathic acute facial paralysis, was first identified by Charles Bell in 1830 as the main cause of sudden facial muscular weakness on one side [1]. Most of the time, the cause of BP is idiopathic. It commonly impacts both sexes equally, displaying a slight inclination towards females [2]. Proposing a connection between unexplained facial paralysis and pregnancy, Sir Charles Bell indicated a higher occurrence of Bell's palsy during pregnancy and the postpartum phase."[3].

Bell's palsy is a peripheral facial palsy that results in facial impairment due to facial nerve inflammation, and its aetiology is unknown. Bell's palsy affects about 25 out of every 100,000 people annually, and although its exact cause is unknown, it is generally thought to have a viral origin [4]. The association between pregnancy and occurrences of Bell's palsy has been established, with a prevalence of 45 cases per 100,000 pregnant women. Notably, the majority of Bell's palsy cases in pregnant women tend to manifest during the third trimester and the postpartum period [3].

When a specific cause cannot be identified, the diagnosis is established by excluding other potential factors. It is defined by the sudden and spontaneous onset of facial nerve weakness or paralysis [5,1]. Facial nerve branches are usually affected by weakness, which can result in altered facial expressions, difficulties closing the mouth and eyelids, and speech impairment. The precise cause of Bell's palsy is yet unknown. Nonetheless, it is frequently linked to the geniculate ganglion experiencing a resurgence of the herpes simplex virus (HSV), which causes oedema and inflammation surrounding the nerve as well as reduced blood supply to the portion of the facial nerve located in the temporal bone [2,6]. Even though it usually goes away independently, up to 30% of patients recover partially, resulting in different degrees of muscle weakness, elevated muscle tone, and synchronous, involuntary movements [7,8].

While the precise cause of facial nerve palsy during pregnancy is unknown, there are several potential causes, including vascular issues, viral infections, and idiopathic causes. Because of the profound physiological changes that take place during pregnancy and the need to safeguard both the mother's and the fetus's health, this condition is an appealing topic for research and clinical observation.

This case study aims to enhance our comprehension of the challenges and intricacies associated with facial nerve palsy in individuals during pregnancy and highlight the crucial role of healthcare providers in achieving the best possible results for both the expectant mother and her unborn child.

## Case presentation

**Figure 1:**
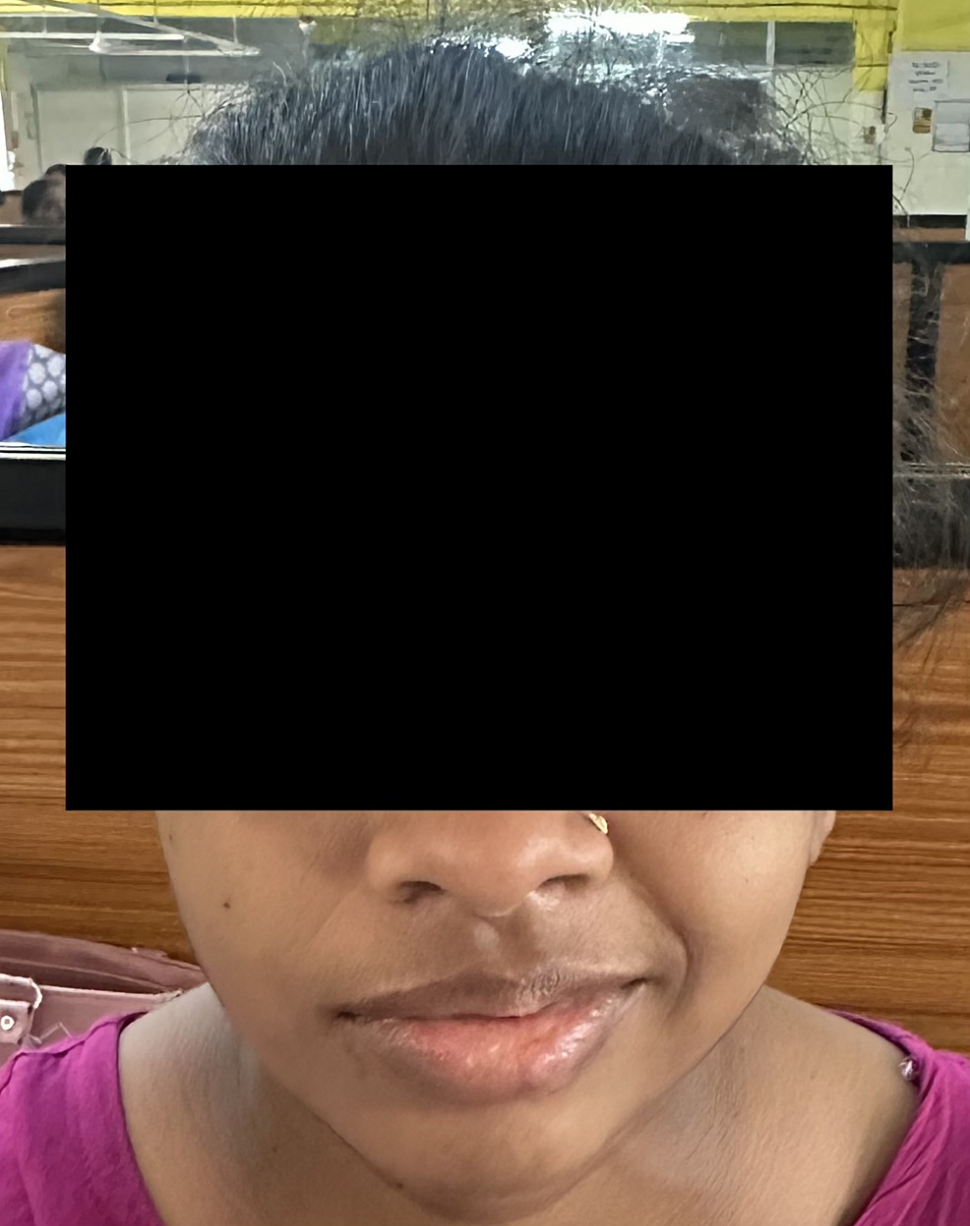
Left-sided facial nerve palsy in the pregnant patient

A 29-year-old primigravida presented to the Obstetrics and Gynaecology outpatient clinic with the complaint of amenorrhoea for eight months; at 32 weeks of gestation, she experienced a sudden onset of weakness on the left side of her face. She was a known case of sickle cell trait (autosomal recessive (AS) pattern). She had an otherwise unremarkable medical history with no history of chronic medical conditions. Her prenatal care had been progressing without any complications until she had the recent development of her facial symptoms for one day. She experienced an abrupt onset of weakness on the left side of her face and encountered challenges in fully closing her left eye, which led to eye dryness. She also noted pronounced drooping on the left side of her mouth. These symptoms had been present for the past 24 hours. Importantly, she did not report any other neurological deficits or systemic symptoms. On examination, she displayed evident facial asymmetry with incomplete eye closure (lagophthalmos), an inability to maintain complete mouth closure and pronounced drooping of the left side of her mouth. Altered facial expressions on the left side were also observed (Figure 1).

**Figure 2:**
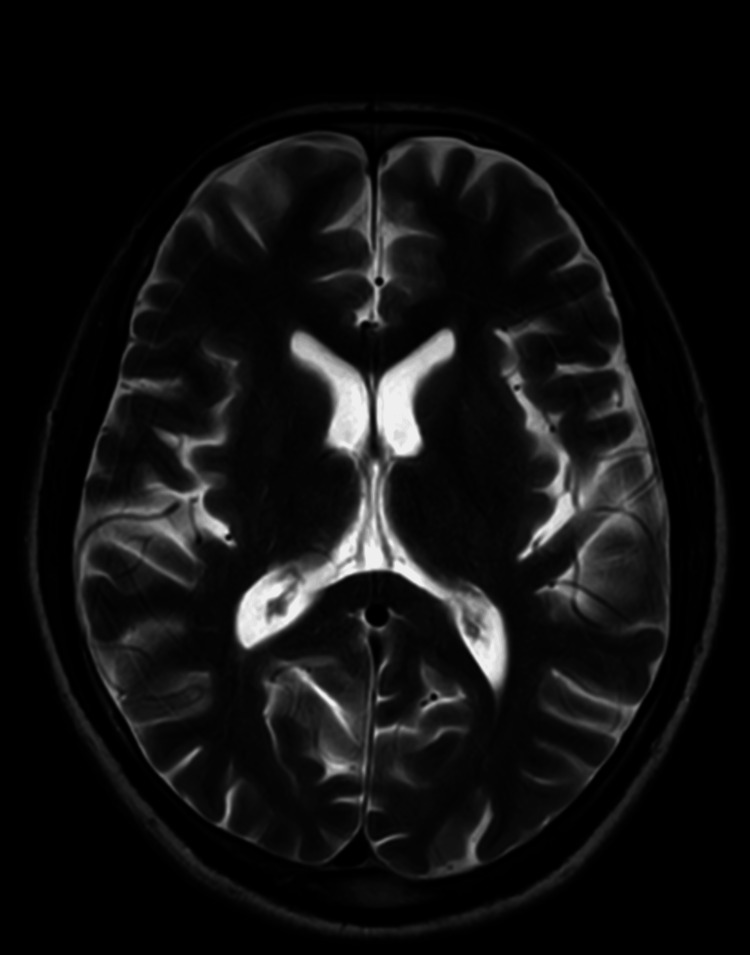
MRI brain plain suggestive of no structural abnormalities or lesions affecting the facial nerve

Vital signs were within normal ranges. In addition to a neurology consultation to further investigate the facial nerve palsy, she underwent an MRI, which showed no structural abnormalities or lesions affecting the facial nerve (Figure 2).

**Figure 3:**
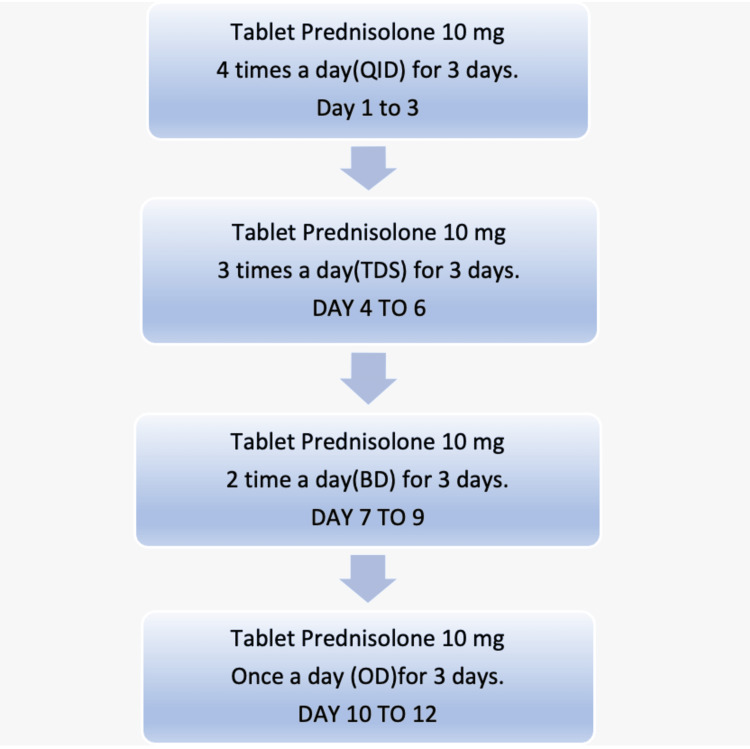
Steroid dosage schedule for the management of facial nerve palsy in pregnancy

Comprehensive blood tests, including serological evaluations for viral infections, were conducted. An ophthalmology consultation was requested to assess her ocular health, and a neurophysiology consultation was sought to perform nerve conduction studies and electromyography. The prescribed dosage schedule included tablet prednisolone at 10 mg four times daily for the initial three days, followed by three times daily for the subsequent three days, then reduced to twice daily for another three days, and finally, once daily for the last three days (Figure 3).

Along with this medication, the physician advised neurophysiotherapy, which was done regularly, twice a day. The ophthalmologist prescribed hydroxypropyl methylcellulose ophthalmic gel 0.3% at night time. The patient's symptoms reduced significantly after starting steroids (prednisolone). She underwent LSCS (lower segment cesarean section) and delivered a healthy baby of 2.6 kg. Her condition steadily got better. Her symptoms completely resolved with the use of tablet prednisolone along with neurophysiotherapy. She was counselled and informed about the idiopathic cause of this condition, and she was discharged from the hospital. Her condition remained stable during her hospital stay, and she received the required multidisciplinary care and approach. It was acknowledged that facial nerve palsy during pregnancy is a relatively rare clinical entity with multifactorial potential causes. The need for a multidisciplinary approach was paramount to ensure optimal care for the pregnant woman and the developing fetus. 

## Discussion

Facial nerve palsy occurring during pregnancy and postpartum constitutes a distinctive clinical phenomenon. It is crucial to adopt a personalized management strategy that prioritizes the safety of the mother and the baby while addressing the mother's fundamental physical requirements. While conventionally thought to be more frequent in pregnancy and the postpartum phase, an increasing body of evidence implies that this might not hold. In a foundational study, Hilsinger et al. exhibited a 3.3-fold higher occurrence of Bell's palsy in pregnancy compared to non-pregnant women [3]. Follow-up research studies indicated a reduced event in the early stages of pregnancy, with a surge of two to four times in frequency observed during the last trimester and the immediate post-delivery period [7]. However, a recent cohort study conducted nationwide in Korea found that the general incidence of Bell's palsy during pregnancy was 0.031%, as opposed to 0.058% in control groups matched for age, gender, place of residence, and income [9].

The clinical depiction of Bell's palsy during pregnancy closely resembles its presentation in non-pregnant individuals. Typical symptoms encompass a drooping eyebrow, flattened nasolabial folds, reduced wrinkles on the affected side, a downturned corner of the mouth, and an inability to perform actions like wrinkling the forehead, displaying teeth, whistling, raising eyebrows, or pursing lips. Challenges in complete eye closure, coupled with an upward movement of the eyeball, leading to the exposure of a portion of the white sclera, are identified as what is commonly known as Bell's sign. Additionally, individuals with Bell's palsy may encounter issues such as dryness in the eye, difficulties in salivation, heightened sensitivity to noise, and changes in taste perception in the anterior two-thirds of the tongue.

About 15% of expectant individuals experiencing abrupt facial paralysis may attribute the condition to underlying factors distinct from Bell's palsy [10]. A thorough evaluation, incorporating a detailed medical history and examination, is essential for refining potential differential diagnoses. Bell's palsy is often mistaken for a stroke because of its sudden onset, presenting as numbness and loss of muscle control on the affected side. However, key distinctions facilitate differentiation: upper motor neuron (UMN) lesions, like strokes, typically spare the upper third of the face, while lower motor neuron (LMN) lesions seen in Bell's palsy lead to complete facial paralysis.

In the final trimester of pregnancy, the immune system faces heightened compromise, primarily due to increased cortisol levels. This weakened immune condition has the potential to trigger the activation of the herpes virus located in the geniculate nucleus of the facial nerve. The resultant activation initiates an inflammatory response, causing direct harm to the nerves through demyelination. Given the notable occurrence of Bell's palsy cases in the third trimester, it is reasonable to consider viral infection as a potential factor [11].

In specific cases of herpes zoster (HZ) infections, the presence of vesicles may not be apparent. It is currently recognized that herpes can play a role in facial palsies, accounting for approximately one-third of cases previously considered idiopathic [12]. Furthermore, reactivation of the oral HSV has been noted in connection with the administration of epidural or intrathecal morphine. As a result, certain instances of postpartum Bell's palsy may be linked to the management of anaesthesia [13].

The expansion of plasma volume and venous stasis induced by pregnancy increases interstitial fluid and peripheral oedema. This can lead to nerve compression in confined spaces, such as carpal tunnel syndrome [11]. In the fallopian canal, the facial nerve experiences mechanical compression resulting from tissue oedema. This hypothesis aligns with the observation that the peak incidence of Bell's palsy often corresponds with the gestational age when interstitial fluid reaches its maximum, typically occurring in the third trimester [14,15]. Following delivery, the restoration of plasma volume surpasses that of interstitial fluid volume, leading to venous congestion and oedema within the enclosed fallopian canal.

The rise in clotting factors throughout pregnancy triggers an intensified coagulative state, potentially resulting in thrombosis of the vasa nervorum. This thrombosis has the potential to impede blood supply, leading to ischemic damage to the facial nerve [13]. Furthermore, some hypotheses propose that the presence of estrogen and progesterone may play a role in the development of Bell's palsy among pregnant individuals [16]. Certain researchers have also discussed a familial predisposition to idiopathic facial nerve conditions [15,17].

The common approach to managing Bell's palsy usually includes a blend of topical eye care, corticosteroids, and/or antiviral medications, with surgical intervention being a seldom-considered option. Steroid treatment has demonstrated effectiveness in facilitating recovery in certain instances [18,19], although there exist varying perspectives on the essentiality of steroid therapy [20,21]. A notable incidence of spontaneous recovery has been noted during the postpartum period, attributed to elevated levels of endogenously produced steroids during pregnancy, the relatively youthful age of the affected individuals, and the resolution of physiological and anatomical changes following childbirth. However, it is not advisable to induce delivery electively to improve Bell's palsy outcomes or prevent pre-eclampsia, as this can lead to premature birth and an increased likelihood of cesarean section. Elective induction should be reserved exclusively for obstetric indications.

Additional research is necessary to examine the potential impact of medications like magnesium sulfate, commonly employed in pre-eclampsia, on the recovery from Bell's palsy [22]. A comprehensive assessment of pregnant women experiencing Bell's palsy is crucial, as it could precede pre-eclampsia, posing substantial risks for both maternal and fetal well-being. Consistent obstetric follow-up is also advised for individuals in this category.

## Conclusions

In summary, this case study elucidates the specific aspects of Bell's palsy within the unique context of pregnancy, revealing the intricacies of facial nerve paralysis during this critical period. A comprehensive diagnostic approach is needed because of the complex nature of this illness, which involves immune system alterations, mechanical compression, and viral reactivation. The management strategies discussed, encompassing topical eye care, corticosteroids, and antiviral medications, provide valuable insights into the treatment landscape for pregnant individuals facing Bell's Palsy. Additionally, the noteworthy rate of spontaneous recovery postpartum further emphasizes the dynamic interplay of factors contributing to the condition. This case report adds to the growing body of knowledge, seeking to enhance comprehension and treatment strategies for Bell's Palsy in pregnant women as we navigate the intricate interplay between the physiological changes of pregnancy and the onset of Bell's Palsy. Ongoing research and additional case studies will further refine our approaches, ultimately resulting in improved outcomes for the management of Bell's Palsy during pregnancy.
